# Mortality in SARS-CoV-2 Hospitalized Patients Treated with Remdesivir: A Nationwide, Registry-Based Study in Italy

**DOI:** 10.3390/v14061197

**Published:** 2022-05-31

**Authors:** Pierluigi Russo, Evelina Tacconelli, Pier Paolo Olimpieri, Simone Celant, Antonietta Colatrella, Luca Tomassini, Giorgio Palù

**Affiliations:** 1 Italian Medicines Agency, Via del Tritone 181, 00187 Rome, Italy; p.olimpieri@aifa.gov.it (P.P.O.); s.celant@aifa.gov.it (S.C.); a.colatrella@aifa.gov.it (A.C.); l.tomassini@aifa.gov.it (L.T.); 2 Infectious Diseases, Department of Diagnostic and Public Health, University of Verona, 37129 Verona, Italy; evelina.tacconelli@univr.it

**Keywords:** remdesivir, COVID-19, mortality, RWE

## Abstract

Remdesivir is the first drug approved for treatment of COVID-19 but current evidence for recommending its use for the treatment of moderate-to-severe disease is still controversial among clinical guidelines. We performed a nationwide, registry-based study including all Italian hospitalized patients with COVID-19 treated with remdesivir to assess the impact of major confounders on crude 15-day and 29-day mortality. Mortality was calculated using the Kaplan–Meier estimator and the Cox proportional-hazards model was applied to analyze the risks by patient’s baseline features. In total, 16,462 patients treated with remdesivir from 29 October 2020 to 17 December 2020 were entered in the study. Crude 15-day and 29-day mortality were 7.1% (95% CI, 6.7–7.5%) and 11.7% (95% CI, 11.2–12.2%), respectively. Being treated within two days of admission reduced the risk of death by about 40% (HR 1.4, 95% CI, 1.2–1.6). Results from the largest cohort of remdesivir-treated patients suggests that mortality in SARS-CoV-2 hospitalized patients is substantially influenced by the days between SARS-CoV-2 diagnosis and drug prescription. Current recommendations and future clinical trials for remdesivir alone or in combination should carefully consider the target population and timing for best efficacy of treatment.

## 1. Introduction

Remdesivir is an adenosine analogue with broad antiviral activity originally proposed as a treatment for Ebola and later repurposed as a potential COVID-19 treatment after its activity against the RNA-dependent RNA polymerase (RdRp) of SARS-CoV-2 was reported [1,2]. The first randomized clinical trial (RCT) on remdesivir efficacy, published in April 2020, assessed 158 patients with SARS-CoV-2 pneumonia and requiring oxygen therapy randomized to remdesivir. The drug was not statistically associated with a difference in time to clinical improvement; however, the trial was not sufficiently powered to detect assumed differences in clinical outcomes [3]. The Adaptive COVID-19 Treatment Trial 1 (ACTT-1), a randomized, double-blind, placebo-controlled trial, published in late 2020, showed remdesivir to be superior to placebo in shorting the time to recovery in hospitalized adults with COVID-19, including also patients in no need of oxygen therapy (18% of the overall assessable population treated with remdesivir) [4]. Mortality by day 29 was 4% and significantly different among patients with a baseline ordinal score of 5 (i.e., hospitalized patients with pneumonia and requiring supplemental low-flow oxygen therapy), and resulted in a 70% decrease in the risk of death compared to placebo [4]. Following the preliminary reports of the ACTT-1, remdesivir (Veklury^®^) obtained the emergency use authorization for the treatment of suspected or laboratory-confirmed COVID-19 in hospitalized patients from the U.S. Food and Drug Administration (FDA) in May 2020, and from the European Medicines Agency (EMA) in June 2020. Full approval by the FDA and a conditional marketing authorization by EMA was then granted in October 2020, and July 2020, respectively.

After the market authorization, three RCTs, assessing the effectiveness of remdesivir on the clinical outcome of COVID-19 patients, showed discordant results. The World Health Organization Solidarity trial found no effect on hospitalized patients by overall mortality, initiation of ventilation, and duration of hospital stay [5]. On the other hand, the ACTT-2 showed similar findings to ACTT-1 in terms of mortality by day 28 in the remdesivir plus placebo arm (4.7%; IC 95%, 2.7–8.1%) among patients with a baseline ordinal score of 5 [6]. Finally, the DisCoVeRy trial showed no effect on 28-day mortality for remdesivir in patients who were admitted to the hospital for COVID-19 symptoms for more than 7 days and requiring oxygen support [7].

To date, the last released guidelines from the European Society of Clinical Microbiology and Infectious Diseases (ESCMID) on the treatment of COVID-19 applied a meta-analysis approach, showing that remdesivir was not associated with an effect on mortality by day 29 (RR 0.76; 95% CI, 0.57–1.02) while decreasing the need for invasive mechanical ventilation or ECMO in hospitalized patients (RR 0.57; 95% CI, 0.42–0.79) [8]. Based on this evidence, the expert panel released a conditional recommendation for the use of remdesivir in COVID-19 hospitalized patients not requiring mechanical ventilation or ECMO (moderate quality of evidence). The same conclusions have been achieved by the Infectious Diseases Society of America (IDSA) [9]. Conversely, the WHO guidance document recommends against the use of remdesivir [10].

A recent meta-analysis (last search on May 2021), which including 11 studies (case series, non-randomized, and randomized controlled trial) assessing clinical trials and the compassionate use of remdesivir, showed that clinical improvement and mortality rates in hospitalized adult patients with moderate-to-severe COVID-19 treated with remdesivir varied strongly across studies [11]. The authors concluded that the evidence is therefore insufficient to confidently recommend the use of remdesivir alone for the treatment of moderate-to-severe COVID-19. After the publication of the meta-analysis a retrospective multicenter American study including 2344 US veterans hospitalized with COVID-19 showed that remdesivir therapy was not associated with improved 30-day survival but was related to a significant increase in the median time to hospital discharge [12].

On 16 December 2021, EMA extended the use of remdesivir [13] to treat early SARS-CoV-2 infection in high-risk patients for disease progression due to the evidence from the PINETREE trial showing that a 3-day course of remdesivir resulted in an 87% lower risk of hospitalization or death than placebo [14].

At the end of November 2021, a new variant of SARS-CoV-2 (B.1.1.529, also called omicron variant) was reported by WHO. Later, in vitro studies showed that several monoclonal antibodies recognizing the spike protein of different variants of SARS-CoV-2 were not able to bind proficiently to the omicron target protein, strongly suggesting a lack of in vivo efficacy against the world dominant variant [15]. On the other hand, remdesivir and the other direct-acting antivirals, targeting conserved viral proteins other than the spike (i.e., RNA-dependent RNA polymerase or the main protease), maintain in vitro activity against the omicron variant [16]. For these reasons, remdesivir is among the few alternative options that might still retain a clinical efficacy on omicron-based SARS-CoV-2 infections.

The aim of this study was to assess the evidence on mortality, progression of disease, and duration of hospital stay from a nationwide, registry-based cohort study of hospitalized patients with COVID-19 treated with remdesivir after adjusting for major epidemiological and clinical features.

## 2. Materials and Methods

The study design was a nationwide, registry-based study including all Italian hospitalized patients with COVID-19 treated with remdesivir from 29 October 2020 to 17 December 2020, corresponding to the second pandemic wave in Italy. Treated patients were not included in the analysis if the required set of data was not complete or erroneous (e.g., missing date of death or implausible date of hospital admission or date of discharge). All patients had at least 29 days of follow-up. Main outcomes are 15-day and 29-day crude mortality, median duration of hospitalization (discharge or death), and the need for intensive or sub-intensive care, and mechanical ventilation or ECMO were also analyzed.

After the decision issued by the European Commission on 3 July 2020 on the granting of a conditional marketing authorization for SARS-CoV-2 positive patients affected by pneumonia who require supplemental oxygen, the Italian Medicines Agency (AIFA) granted a reimbursement of remdesivir by the Italian National Healthcare Service (INHS) exclusively to the subgroup of patients hospitalized with SARS-CoV-2 pneumonia requiring supplemental low-flow oxygen therapy (but not mechanical ventilation or ECMO) [17]. The remdesivir treatment was provided to the centers for a total duration of treatment of up to 5 days (200 mg infusion on the first day, followed by 100 mg infusion per day). All patients undergoing treatment with remdesivir were mandatorily included in the national registry since 29 October 2020. The AIFA Monitoring Registry System is a web-based national platform that was introduced in 2005. It is aimed at monitoring predefined treatments in specific clinical conditions (generally high-cost and priority medicines), for which the reimbursement by the INHS is granted. Data collected by monitoring registries are currently used to inform national regulation of patient access and to provide evidence on medicine use in the real-world context [18,19]. The registry collects data on baseline characteristics of patients, hospital admission and discharge, the prescription of remdesivir, the dispensation of vials to the hospital ward, and patients’ treatment outcome (expressed as the duration of hospitalization or mortality).

In order to face concerns on data completeness, with special reference to the under-reporting of patient deaths, and to enhance data accuracy, death dates of patients included in the registry were also obtained from the national register office for the resident population (ANPR), which is a central database maintained by the Ministry of the Interior of Italy (decree 82/2005, art. 62). By Italian Laws, death certificates must be inserted in the ANPR within 10 days from the event. Therefore, data cut-off and latest administration date were selected to minimize the number of non-recorded deaths. According to Italian laws, the current study does not require any formal approval from ethical committees.

### Statistical Analysis

Data were extracted using SQL scripts from an oracle database. Crude 15-day and 29-day mortality were calculated using the Kaplan–Meier estimator considering all events from day 1 (treatment start) to 14 or 28 and censoring events observed at day 15 and 29 [4]. The Cox proportional-hazards (PH) model was applied to analyze the risks connected to patients’ baseline features relative to all-cause mortality by day 29 in patients with 5 days or less from hospital admission to drug prescription (DAP). This cut-off was selected to minimize the risk of including patients that were hospitalized also for non-COVID-19 related reasons. PH assumption was tested by checking for non-zero slopes of coefficients via Schoenfeld residuals. All variables have been used as categorical variables; age has been stratified as reported in Beigel et al. [4]; the adjusted attack rate has been defined as in Riccardo et al. [20]; and DAP was stratified in four classes: 0–2 days, 3–5 days, 5–9 days, and 10 days or more. Cox-adjusted survival curves were obtained for all the variables adopted in the Cox model. In particular, a survival curve was predicted for every subject of our census; the aggregated survival curve was obtained as an average (over all time points) of the single curves. Different Cox models were generated using all significant characteristics (age, gender, days since hospital admission, and adjusted attack rate) and stratifying for the variable of interest [21].

## 3. Results

During the study period, 16,999 Italian patients hospitalized for COVID-19 pneumonia requiring supplemental low-flow oxygen therapy (but not mechanical ventilation or ECMO) were eligible for remdesivir treatment according to the criteria and recommendations established by AIFA CTS [17], and were registered in the AIFA Monitoring Registry System. Among them, 537 patients (3.2%) were not included in the analysis because of missing or inadequate data (uncertainty about survival status/date of death, erroneous reported admission, and prescription or discharge dates). In total, 16,462 patients had 29-day follow-up data and were included in the analysis. The mean age (±SD) of the population was 66.3 years (±13.7) with a male/female ratio of 2/1. In most of the patients, remdesivir was prescribed within 3 days since hospital admission (13791 cases; 83.8%), for a median duration of treatment of 5 days. Patients with 10 or more DAP (288; 1.7%) were probably admitted in the hospital for non-COVID-19 related causes and then tested positive after showing symptoms. In total, 75% (*n* = 12371; adjusted attack rate high-to-intermediate) of patients were admitted in centers located in regions that were strongly affected by the first pandemic wave. Baseline characteristics of the population are described in Table 1.

### 3.1. Crude Mortality and Cox-Adjusted Estimates

**Figure 1:**
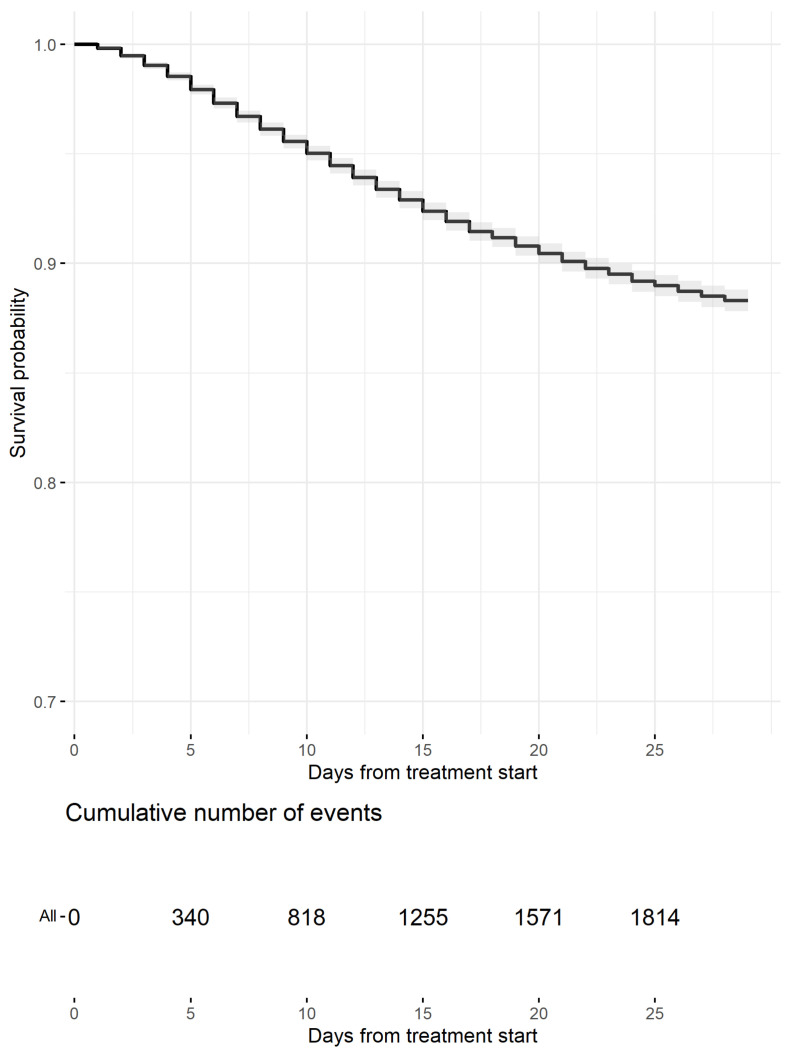
Kaplan–Meier Estimates of survival probability up to day 29 (events are censored at day 29). The gray area represents the 95% confidence band. Table of cumulative events (deaths) is reported below the curve.

**Figure 2:**
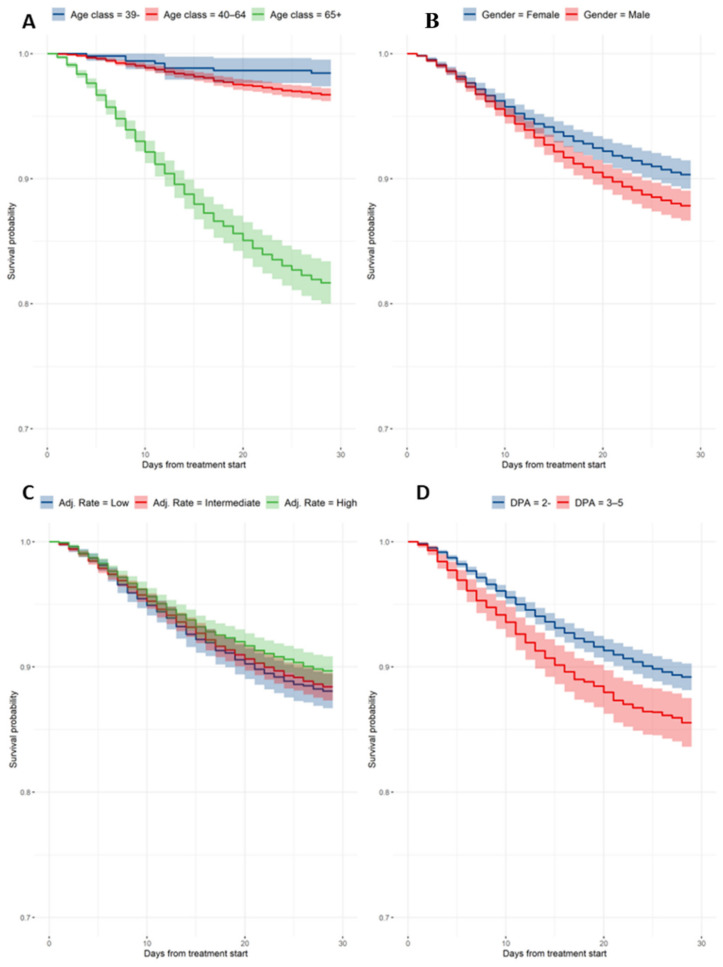
Cox-adjusted survival curves. (**A**) Estimates by age group; (**B**) estimates by gender; (**C**) estimates by adjusted attack rate; and (**D**) estimates by days from hospital admission.

At data cut-off, 2277 deaths (13.8%) were registered. The Kaplan–Meier estimates by 15-day and 29-day mortality were 7.1% (1168 deaths; 95% CI, 6.7–7.5%) and 11.7% (1925 deaths; 95% CI, 11.2–12.2%), respectively (Figure 1 and Table 2). Hazard ratios (HRs) from the Cox models and mortality estimates from the adjusted survival curves are reported in Table 2 and Figure 2. Significant features in explaining mortality included age class, gender, adjusted attack rate, and DAP. In particular, having two DAP or less reduced the risk of death by 39% compared to having three to five DAP.

Cox-adjusted estimates were calculated for all relevant baseline features (Table 2): 29-day mortality by age group was 1.5% (95% CI, 0.5–2.6%), 3.3% (95% CI, 2.8–3.8%), and 18.3% (95% CI, 16.6–20.0%) for patients less than 40 years old, between 40 and 64 years old, and 65 years old or older, respectively. Estimated female mortality by day 29 was 9.7% (95% CI, 8.5–10.8%), significantly lower compared to the male mortality by day 29, which was 12.2% (95% CI, 11.0–13.3%; HR male vs. female: 1.3). Mortality by day 29 was 10.8% (95% CI, 9.7–11.9%) for patients with zero to two DAP and 14.5% (95% CI, 12.5–16.4%) in the case of three to five DAP. Mortality estimates for the adjusted attack rate by day 29 were 11.9% (95% CI, 10.6–13.3%), 11.6% (95% CI, 10.5–12.7%), and 10.3% (95% CI, 9.2–11.5%).

### 3.2. Hospitalization and Need of Mechanical Ventilation

Data on hospital discharge and the need for mechanical ventilation were available in 12,785 (77.7%) and 12,086 (73.4%) patients, respectively. The median duration of hospitalization (interquartile range) after starting treatment with remdesivir was 13 days (8 to 20 days). In total, 3556 cases out of 12,086 (29.4%) went through the sub-intensive or intensive care ward before either hospital discharge or death; 695 patients (5.8%) required mechanical ventilation or ECMO.

## 4. Discussion

Our study, to the best of our knowledge, is the largest cohort assessing adjusted mortality in hospitalized patients with SARS-CoV-2 pneumonia and requiring supplemental low-flow oxygen therapy. Results show significant effects of the days from hospital admission to drug prescription, age, gender, and the attack rate on 29-day mortality.

A 39% increase of the risk of death at day 29 was observed in patients with three or more days from hospital admission to remdesivir prescription when compared to those having a shorter interval. This result appears to be in line with the recent evidence from the GS-US-540-9012 (PINETREE) trial, which showed a lower risk of hospitalization or death in non-hospitalized patients receiving remdesivir treatment for a 3-day course within 7 days of symptoms onset compared to placebo [14].

The estimate of 29-day mortality after remdesivir treatment in Italy was equal to 11.7%, higher than that observed in the ACTT-1 and ACTT-2 RCTs [4,6]. This might be due to the older age of patients enrolled in the AIFA’s registry (66.3 years on average) than that recorded in the ACTT-1 trial (58.6 years on average). In total, 45.4% (*n* = 7466) of Italian patients were less than 65 years old, against the 65.4% of ACTT-1 trial patients [4]. Indeed, results in our cohort show a substantial effect of age on mortality, with older patients (≥65 years) treated with remdesivir having a near 13 times higher death risk than those treated with the same drug aged less than 40. The overall mortality after remdesivir treatment in Italy appeared to be higher also than that observed in the WHO Solidarity trial (i.e., 9.4% in the subgroup of patients not requiring ventilation at randomization) [5]. It is worthy to note that the inclusion criteria of the WHO trial were different to those used for patients’ eligibility in the AIFA’s registry and the proportion of patients aged less than 70 years old was 81.8% and 57.5% in the WHO Solidarity trial and AIFA registry, respectively. Moreover, 29-day mortality among patients with moderate COVID-19, largely composed of hospitalized patients requiring supplemental oxygen (92%), was found to be around 6% in the DisCoVeRy trial [7]; the median age in the overall remdesivir group was 63 years but the ordinal scale-stratified median age was not reported to draw a fairer picture of our data.

Our study underlines the difference in mortality rates between RCTs and the real-life use of remdesivir. In a Denmark observational study including also very severe COVID-19 patients (29% of patients on mechanical ventilation or ECMO), the mortality rate at day 14 was substantially higher than that reported in the AIFA registry (i.e., 24.6% in the weighted population with a mean age of 67.1 years) [22]. Despite a similar mean age (66.6 vs. 66.3 years old), in a study on U.S. Veterans first admitted to the Veterans Health Administration (VHA) acute care setting during the remdesivir EUA with a PCR-positive test for SARS-CoV2 within 14 days before or during hospitalization [12], counting 26.6% of remdesivir-treated patients in ICU or mechanical ventilation at inclusion, mortality at day 30 was found to be 12.4% after propensity score-matching, which was in line with that estimated for male patients (12.1%) in the AIFA registry that, however, was less severe. Differences between the real-world evidence and RCTs might be due to external and internal factors such as the case-mix of patients, the distribution or exclusion of comorbidities, late diagnosis, and the variation in the distribution of viral variants.

It is important to note that the limits of reimbursement set by the AIFA Scientific Committee on October 2020 allowed for the remdesivir treatment in a patient subgroup which exactly corresponded to those recommended currently by ISDA and ESCIMD guidelines, which provide a conditional recommendation for adopting remdesivir in patients on supplemental oxygen but not on mechanical ventilation or ECMO, and suggest treatment with five days of remdesivir rather than 10 days of remdesivir.

Our study also registered a 28% higher risk of death among males than among females treated with remdesivir. This finding was also observed in a previous population-based cohort of hospitalized Italian patients performed after the first pandemic wave and before the introduction in Italy of remdesivir [23]. However, the higher COVID-19 mortality risk in males over females appears to be consistent overall with sex-based differences in hormonal and immunological responses, which affect SARS-CoV-2 entry in human cells [24].

A small effect on mortality of the adjusted attack rate was registered. The age-adjusted attack rate per a population of 100,000 was used during the first pandemic wave in order to aggregate Italian regions with similar transmission patters during the COVID-19 pandemic [20]. According to this ranking, patients hospitalized in Italian regions having higher attack rates during the first pandemic wave displayed a −15% lower mortality risk than patients in regions having a low attack rate. This result could be the consequence of the transformative changes of the management of COVID-19 patients (especially those in need of intensive care), where the impact of the first pandemic wave was more relevant, which increased the experience of health professionals and their organization, improving the overall effectiveness of the health care [25].

Some limitations of this study are represented by the absence of information on comorbidities, on the time span between symptoms’ onset and remdesivir treatment, concomitant treatments during hospitalization, and the lack of a control group of patients not treated with remdesivir. However, the distribution of comorbidities would not directly impact the start of therapy; it would be plausible that patients with severe comorbidities would have been treated earlier than patients with less severe or no comorbidities [26]. Regarding concomitant therapies, we expect that almost all patients in our cohort also received corticosteroid treatment based on national and regional recommendations for treatment of COVID-19 patients [27]. Furthermore, another possible limitation might be represented by the observation window, which covers the second Italian pandemic wave from October 2020 up to January 2021. This decision was made to minimize the risk of bias in the analysis of mortality, which could have been affected by the interaction of several factors, regardless of remdesivir efficacy in the real-world practice, including the evolving disease severity of COVID-19 related to the emergence of new variants of concern, the burden on healthcare facilities and hospitals, and the population’s vaccine coverage. Monoclonal antibodies for COVID-19 could, in turn, have affected the mortality rate; in Italy, they were introduced after March 2021.

In conclusion, this study suggests that mortality in SARS-CoV-2 hospitalized patients treated with remdesivir is substantially influenced by the days between SARS-CoV-2 diagnosis and drug prescription. Significant effects of age and gender were also observed, however, they were registered before remdesivir was available in Italy [23] and therefore not necessarily attributable to the treatment. Furthermore, the manuscript provides additional cues to further investigate the effect of remdesivir in the early stage of the infection and as soon as possible after symptoms’ onset. Current recommendations and future clinical trials for remdesivir alone or in combination should carefully select the target population and timing for best efficacy of treatment.

## Figures and Tables

**Table 1:** Baseline characteristics of 16,462 patients treated with remdesivir in Italy from 29 October 2020 to 17 December 2020.

Characteristics	*n* (%) 16,462
Gender, female	5430 (32.99%)
Gender, male	11,032 (67.01%)
Age, median (1st–3rd q)	66.87 (56.78–76.81)
Age, mean (sd)	66.34 (13.70)
Age, 39−	541 (3.29%)
Age, 40–64	6925 (42.07%)
Age, 65+	8996 (54.65%)
DAP, 0–2	13,791 (83.77%)
DAP, 3–5	1886 (11.46%)
DAP, 6–9	497 (3.02%)
DAP, 10+	288 (1.75%)
AAR High	4904 (29.79%)
AAR Intermediate	7467 (45.36%)
AAR Low	4091 (24.85%)

Legend: DAP = days between hospital admission and prescription, and AAR = adjusted attack rate.

**Table 2:** Hazard ratios (HRs) from Cox models and mortality estimates from adjusted survival curves.

**Baseline Characteristics**	**HR**	**Low 95% CI**	**High 95% CI**
Gender, M vs. F	1.28	1.16	1.42
Age, 40–64 vs. <40 years	2.14	1.06	4.33
Age, ≥65 vs. <40 years	13.05	6.52	26.16
AAR Intermediate vs. Low	0.97	0.86	1.09
AAR High vs. Low	0.85	0.75	0.97
DAP 3–5 vs. 0–2	1.39	1.22	1.58
**Cox-adjusted mortality by day-15 estimates**	**%**	**Low 95% CI**	**High 95% CI**
<40	1.16	0.23	2.08
40–65	1.83	1.47	2.19
≥65	12.03	10.79	13.26
Sex, F	6.26	5.41	7.09
Sex, M	7.83	6.97	8.68
DAP, 0–2	6.89	6.14	7.62
DAP, 3–5	9.85	8.29	11.38
AAR Low	7.82	6.75	8.87
AAR Intermediate	7.32	6.5	8.12
AAR High	6.8	5.91	7.67
**Cox-adjusted mortality by day-29 estimates**	**%**	**Low 95% CI**	**High 95% CI**
<40	1.54	0.47	2.61
40–65	3.28	2.76	3.79
≥65	18.32	16.61	20.00
Sex, F	9.67	8.53	10.79
Sex, M	12.16	10.96	13.34
DAP, 0–2	10.80	9.73	11.86
DAP, 3–5	14.46	12.50	16.37
AAR Low	11.94	10.55	13.29
AAR Intermediate	11.59	10.47	12.68
AAR High	10.32	9.17	11.45

Legend: DAP = days between hospital admission and prescription, and AAR = adjusted attack rate.

## Data Availability

Data cannot be shared due to legal and privacy issues.
